# Gas entrapment and microbial N_2_O reduction reduce N_2_O emissions from a biochar-amended sandy clay loam soil

**DOI:** 10.1038/srep39574

**Published:** 2016-12-23

**Authors:** Johannes Harter, Ivan Guzman-Bustamante, Stefanie Kuehfuss, Reiner Ruser, Reinhard Well, Oliver Spott, Andreas Kappler, Sebastian Behrens

**Affiliations:** 1 Geomicrobiology & Microbial Ecology, Center for Applied Geosciences, University of Tuebingen, Tuebingen, Germany; 2 Fertilization and Soil Matter Dynamics, Institute of Crop Science, University of Hohenheim, Stuttgart, Germany; 3 Plant Ecology and Ecotoxicology, Institute of Landscape and Plant Ecology, University of Hohenheim, Stuttgart, Germany; 4 Institute of Climate-Smart Agriculture, Johann Heinrich von Thünen-Institut, Federal Research Institute for Rural Areas, Forestry and Fisheries, Braunschweig, Germany; 5 Department of Soil Physics, Helmholtz Centre for Environmental Research – UFZ, Halle/Saale, Germany; 6 Department of Civil, Environmental, and Geo-Engineering, University of Minnesota, Minneapolis, MN, USA; 7 BioTechnology Institute, University of Minnesota, St. Paul, MN, USA

## Abstract

Nitrous oxide (N_2_O) is a potent greenhouse gas that is produced during microbial nitrogen transformation processes such as nitrification and denitrification. Soils represent the largest sources of N_2_O emissions with nitrogen fertilizer application being the main driver of rising atmospheric N_2_O concentrations. Soil biochar amendment has been proposed as a promising tool to mitigate N_2_O emissions from soils. However, the underlying processes that cause N_2_O emission suppression in biochar-amended soils are still poorly understood. We set up microcosm experiments with fertilized, wet soil in which we used ^15^N tracing techniques and quantitative polymerase chain reaction (qPCR) to investigate the impact of biochar on mineral and gaseous nitrogen dynamics and denitrification-specific functional marker gene abundance and expression. In accordance with previous studies our results showed that biochar addition can lead to a significant decrease in N_2_O emissions. Furthermore, we determined significantly higher quantities of soil-entrapped N_2_O and N_2_ in biochar microcosms and a biochar-induced increase in typical and atypical *nosZ* transcript copy numbers. Our findings suggest that biochar-induced N_2_O emission mitigation is based on the entrapment of N_2_O in water-saturated pores of the soil matrix and concurrent stimulation of microbial N_2_O reduction resulting in an overall decrease of the N_2_O/(N_2_O + N_2_) ratio.

Nitrous oxide (N_2_O) is a greenhouse gas that is mainly produced during microbial nitrogen transformation processes in natural and agricultural soils. In 2011, atmospheric N_2_O concentration was at 324 ppb, which is an increase of 20% in comparison to 1750^1^. Since N-fertilization provides the substrates for N_2_O production in soils atmospheric N_2_O concentrations have increased together with the intensification of agricultural practices. The rising demand for food and the reliance of agriculture on nitrogen fertilizers will likely further increase N_2_O emissions in the future^2^. In order to counteract this trend, strategies to mitigate N_2_O emissions arising from current agricultural practice are urgently required.

Among other strategies, biochar application to soils has been discussed as a potential option to mitigate N_2_O emissions from agricultural soils^3^,^4^. Biochar is a carbon-rich solid, produced with the intent to be used as soil amendment. It is produced by thermal decomposition of organic material under low oxygen conditions. Recently biochar has gained a lot of attention due to its potential to not only improve soil quality but also serve as a potent climate change mitigation strategy^5^,^6^,^7^. For example, it has been demonstrated that due to its physicochemical properties biochar can improve plant growth while simultaneously sequestering atmospheric carbon dioxide^8^,^9^. Although biochar’s physicochemical properties vary strongly with the type of biomass used as feedstock and the charring conditions during thermal decomposition, most biochars share common characteristics. The majority of biochars have a highly aromatic carbon structure, a neutral to alkaline pH, and a large surface area^6^,^10^. It has been shown in a recent meta-analysis that soil biochar amendment can significantly mitigate soil-derived N_2_O emissions^11^,^12^. In addition, various studies revealed biochar effects on functional microbial guilds involved in nitrogen transformation processes such as nitrification and denitrification^13^,^14^,^15^,^16^,^17^.

Microbial nitrification and denitrification are thought to be the main pathways for N_2_O production in natural and agricultural soils^18^. Even though both processes are considered to account for the majority of soil-derived N_2_O emissions, other microbial processes such as dissimilatory nitrate reduction to ammonium (DNRA) or co-denitrification have been assumed to contribute significantly to total N_2_O emissions under specific environmental conditions^19^,^20^. Oxygen partial pressure, which is largely controlled by the soil water content and aerobic respiration, and the availability of nitrogen compounds represent key factors controlling N_2_O production and release^18^. The highest N_2_O emissions often occur in situations when oxygen is limiting and mineral nitrogen contents are high, for example after heavy rainfall on fertilized soils. Although high water contents (up to total saturation) in most soils in temperate regions are usually temporary, it has been shown that the amount of N_2_O emitted under these conditions accounts for a high proportion of the total annual N_2_O emissions^21^,^22^. Low oxygen partial pressure promotes anaerobic nitrogen transformation processes such as denitrification, which is considered as the prevailing N_2_O producing process when the water-filled pore space (WFPS) reaches 60–90%^18^. Denitrification is performed by many facultative and strict anaerobic chemoorganotrophic bacteria and describes the stepwise enzymatic reduction of nitrate (NO_3_^−^) to dinitrogen (N_2_)^18^,^23^. Obligate intermediates are nitrite (NO_2_^−^), nitric oxide (NO) and N_2_O. The enzymes catalysing the reduction reactions are encoded by the functional genes *narG* and *napA* (nitrate reductases), *nirK* and *nirS* (nitrite reductases), *norB* (nitric oxide reductase) and *nosZ* (nitrous oxide reductase)^23^,^24^. As some denitrifiers lack a functional *nosZ* gene and nitrous oxide reductases are highly oxygen and pH sensitive, the last step of denitrification, N_2_O reduction to N_2_, is often impaired and N_2_O is released as the denitrification end product^25^,^26^,^27^. Although N_2_O can be produced by several nitrogen transformation processes, the only known dominant sink for N_2_O is the microbial reduction to N_2_ via *nosZ*-encoded nitrous oxide reductases^28^. Hence, the quantity of N_2_O emitted from soils depends substantially on the activity of N_2_O-reducing microorganisms as the enzymatic reduction of N_2_O to N_2_ directly controls the N_2_O/(N_2_O + N_2_) emission ratio. For a long time N_2_O reduction was thought to be a functional trait restricted to ‘classical’ denitrifiers. In addition to the *nosZ* gene ‘classical’ denitrifiers usually also harbour other denitrification genes such as *nirK* and *nirS* and most of them are affiliated with the Proteobacteria^23^,^29^. However, recent studies have provided evidence for the existence of a new clade of N_2_O reducers containing phylogenetically and physiologically more diverse microorganisms^30^,^31^. These microbes carry an atypical form of the *nosZ* gene and about half of them do not carry a nitrite reductase gene. Thus they are not ‘classical’ denitrifiers and many of them only have the genetic capability to perform the last step of denitrification, i.e. the reduction of N_2_O to N_2_^30^,^31^,^32^,^33^. Independently of the dominating microbial N_2_O production pathway, a high amount of water-saturated soil pores also directly affects diffusion of N_2_O molecules from the site of production to the soil surface^34^. When compared to air, the diffusion coefficient of N_2_O is approximately 4 orders of magnitude lower in water^35^. Consequently, N_2_O can be retained in the water-filled pores for several days while slowly diffusing through the network of soil pores towards the soil surface which can lead to enhanced N_2_O reduction activities due to the long residence time of N_2_O in soil^34^,^36^,^37^. This retardation process has been described as gas “entrapment” in previous studies and occurs frequently in soils with elevated water content^34^.

According to recent studies that reported lower N_2_O emissions from biochar-amended soil likely explanations for the suppression of N_2_O emission are (i) a biochar-induced decrease in denitrification activity resulting in lower N_2_O production rates^38^,^39^, (ii) an increase in microbial N_2_O reduction (e.g. by a pH increase) leading to a decreased N_2_O/(N_2_O + N_2_) emission ratio^11^,^15^,^16^,^40^, or (iii) the contribution of abiotic processes such as sorption of N_2_O onto biochar particles and abiotic reduction of N_2_O to N_2_ by redox active compounds^41^,^42^. However, systematic studies focusing on the underlying geochemical and microbial processes causing N_2_O emission mitigation are rare and the contributing mechanisms are not fully understood yet.

In order to further improve our understanding of the underlying processes, we set up soil microcosm experiments with wet soil (90% WFPS) and wood-derived biochar, which were fertilized with a ^15^NO_3_^−^-labelled NH_4_^15^NO_3_ solution. We quantified important soil chemical properties, determined the isotopic signatures of different nitrogen pools and quantified denitrification-specific functional marker genes and transcripts. The main objectives of this study were to determine biochar’s impact (i) on nitrification and denitrification rates, (ii) the contribution of source processes to N_2_O emissions, (iii) NO_3_-derived N_2_O and N_2_ emissions, (iv) N_2_O and N_2_ remaining entrapped in soil, and (v) the abundance and activity of functional microbial guilds contributing to soil denitrification.

## Material and Methods

### Soil and biochar properties

Soil was sampled from the top 10 cm at an urban gardening site of the University of Tuebingen, Germany (48°32′3″N, 9°4′16″E). The soil was classified as an Anthrosol (World Reference Base for Soil Resources, 2014) with 49.8% sand, 25.8% silt, and 24.4% clay (USDA soil texture class: sandy clay loam). Biochar was obtained from Swiss Biochar (Belmont-sur-Lausanne, Switzerland). It was produced by slow pyrolysis (620 °C) of wood residues from a wood chip production plant in accordance with the standards of the European Biochar Certificate (EBC, http://www.european-biochar.org). Important physical and chemical properties of soil and biochar are summarized in Table 1.

### Soil microcosm setup and sampling

In total three similar soil microcosm experiments with slightly different sampling strategies were set up (i.e. experiment 1 to 3) to quantify N_2_O and N_2_ fluxes, soil nitrogen transformation rates and functional denitrification genes and mRNA transcripts.

Prior to microcosm setup, the field moist soil (water-filled pore space (WFPS): 40.6%) sampled from the top 10 cm and the biochar were passed through a 2 mm sieve and thoroughly homogenized. Soil microcosms were set up in 250 ml glass beakers (experiment 1 and 2) or 250 ml wide neck glass bottles (experiment 3). Control microcosms consisted of the field moist equivalent of 100 g dry soil. Biochar microcosms contained the field moist equivalent of 95 g dry soil and 5 g dry biochar, which corresponded to a biochar content of 5% (w/w). Both, control microcosms and biochar microcosms were set up in triplicates. After preparation, all soil microcosms were homogenized with a spatula and compacted by exposing them to 10 blows of a 125 g hammer falling 20 cm. The surface area of the hammer was identical to the soil surface area to ensure homogenous compaction. At beginning of the experiments the soil microcosms were fertilized with a ^15^NO_3_^−^-labelled NH_4_^15^NO_3_ solution (experiment 1: 50 at% ^15^N, experiment 2 and 3: 66 at% ^15^N) at a rate of 332 mg N kg^−1^ (equivalent to 100 kg N ha^−1^ estimated based on the soil surface area in the microcosms). To uniformly distribute the ^15^N label in the soil microcosms the NH_4_^15^NO_3_ solution was spread evenly over the soil surface to allow homogenous infiltration. The amount of water added with the NH_4_^15^NO_3_ solution (control: 36 ml, biochar: 49 ml) was adapted to adjust WFPS to 90%. To allow gas exchange with ambient atmosphere but decrease evaporation rates soil microcosms were covered with a perforated aluminium foil. During incubation WFPS was controlled gravimetrically and held constant by periodically replenishing the evaporated water with deionized water. Soil microcosms were incubated at a constant temperature of 20 °C.

#### Experiment 1: Total and NO_3_^−^-derived N_2_O emissions, soil chemical analyses and qPCR analyses

In experiment 1 sampling took place right after microcosm setup (day 0) and after 1, 2, 4, 7 and 10 days of incubation. We set up 36 soil microcosms to allow destructive soil sampling of a set of 6 microcosms (3 control and 3 biochar microcosms) at each sampling time point. At each time point of sampling we collected soil samples for soil chemical and qPCR analyses as well as gas samples for N_2_O and NO_3_^−^-derived N_2_O quantification from the same set of microcosms.

For the determination of N_2_O and NO_3_^−^-derived N_2_O emission rates (*ER*_*p*_), soil microcosms (250 ml glass beakers) were placed in gas-tight enrichment containers consisting of modified 1.7 l preserving glass jars equipped with luer-lock stopcocks for gas sampling. Gas-tightness was confirmed experimentally prior to experiment start. Enrichment containers were closed and after 1 h of enrichment headspace gas samples were taken and transferred immediately to evacuated 20 ml (N_2_O) and 120 ml (^15^N_2_O) glass vials, capped with butyl rubber septa, using a syringe. In addition, lab air gas samples were taken directly before the containers were closed for gas enrichment, to obtain starting values for N_2_O and NO_3_^−^-derived N_2_O.

Directly after the gas sample was taken (1 h) soil microcosms were removed from the enrichment containers and the entire soil was transferred into clean vessels, homogenized using a spatula and sub sampled for nucleic acid extraction and soil chemical analyses. Sub samples for nucleic acid extraction consisted of the equivalent of 2 g dry soil and were directly frozen at −80 °C.

#### Experiment 2: NO_3_^−^-derived N_2_O and N_2_ emissions

In experiment 2 gas samples were collected repeatedly from six soil microcosms at the same time points as in experiment 1 (days 0, 1, 2, 4, 7 and 10).

For NO_3_^−^-derived N_2_O and N_2_ emission rate (*ER*_*p*_) determination, soil microcosms (250 ml glass beakers) were placed inside enrichment containers as described above. To decrease the high atmospheric N_2_ background (78%) and thus improve ^15^N_2_ quantification^43^,^44^, gas enrichment was performed in an artificial atmosphere. After closure, enrichment containers were purged with an artificial gas mixture consisting of 21% O_2_, 2% N_2_ and 77% He. To remove remaining gases from the headspace and the soil we first applied a vacuum using a pump. When an under-pressure of −700 mbar was reached, containers were filled with the artificial gas mixture until an over-pressure of 300 mbar was achieved. This procedure was repeated 5 times. The last filling was brought to ambient atmospheric pressure by exhausting excess gas through a water-filled vessel.

Headspace samples to analyse ^15^N enrichment of N_2_O and N_2_ by isotope ratio mass spectroscopy were taken 1 and 3 h after purging the enrichment container. For gas sample collection, a 12 ml screw-cap exetainer (Labco Limited, Lampeter, UK), filled with the artificial gas mixture, was connected to the gas enrichment container using a needle. In addition, a syringe (70 ml) was connected to the exetainer with a second needle. Headspace gas was repeatedly pumped through the exetainer into the syringe and back into the headspace of the gas enrichment container by drawing and expelling the syringe completely (70 ml) for 5 times. Preliminary tests confirmed that this procedure ensures equal concentrations in the 12 ml screw-cap exetainer and the enrichment container. All equipment such as tubes, syringes, needles and stopcocks were purged with the artificial gas mixture before sampling and kept closed (tubes, stopcocks) or were sealed using butyl rubber stoppers.

#### Experiment 3: NO_3_^−^-derived N_2_O and N_2_ entrapment in soil

In experiment 3 six soil microcosms (250 ml wide neck glass bottles) were incubated for two days.

In addition to NO_3_^−^-derived N_2_O and N_2_ emission rate (*ER*_*p*_) determination, we also determined the soil entrapment rate (*SER*_*p*_) i.e. the fraction of NO_3_^−^-derived N_2_O and N_2_ produced within 3 h of gas enrichment that remained entrapped in the soil. It has been shown by Holt *et al*.^45^ that destroying the soil matrix by creating and shaking soil slurries and subsequent sampling of the headspace provides an appropriate method to quantify entrapped gases^45^. To allow the creation of a shakeable soil slurry after 3 h of gas enrichment we used 250 ml wide neck glass bottles which served as soil microcosms and enrichment containers instead of 250 ml glass beakers and the enrichment containers described above.

After closure of the bottles using butyl rubber stoppers purging with the artificial gas mixture as well as the gas sampling after 1 and 3 h was performed exactly as described for experiment 2. After the second gas sample was taken (3 h after purging) a defined volume of deionized water was added to create a soil slurry. The resulting over-pressure was set off by exhausting excess gas through a water-filled vessel. The slurry was horizontally shaken on a shaker at 150 rpm for 2 min. Afterwards another gas sample was collected from the headspace as described for experiment 2.

### Soil chemical analyses and gross nitrogen transformation rates

#### Soil and biochar characterization

Properties of the initial soil (Table 1) were determined using standard laboratory methods commonly used in soil sciences. For details please refer to the Supplementary Information. All biochar properties were determined by Eurofins (Eurofins Umwelt Ost, Freiberg, Germany) according to the guidelines given by the European Biochar Certificate (http://www.european-biochar.org).

#### Soil chemical and stable isotope analyses

Soil extracts for chemical analyses were prepared using a 2 M KCL solution directly after soil sub sampling at each time point of sampling (0, 1, 2, 4, 7, 10 days). The equivalent of 5 g dry soil was mixed with 25 mL of 2 M KCL and shaken horizontally for 1 h at 150 rpm. After sedimentation of larger particles (15 min) the supernatant was filtered through a 0.45 μm pore size syringe filter. The obtained filtrate was stored at 4 °C and analysed for NH_4_^+^, ^15^NH_4_^+^, NO_3_^−^, ^15^NO_3_^−^ within 4 weeks. The absence of nitrogen transformation reactions in the filtrates under these storage conditions was experimentally confirmed in preliminary tests.

NH_4_^+^ and NO_3_^−^ contents were determined spectrophotometrically using a segmented flow analyser (AA3 AutoAnalyzer 3 HR, Seal Analytical, Southampton, UK). ^15^N enrichment of NH_4_^+^ and NO_3_^−^ was quantified directly from the filtrate using the SPINMAS technique, as described in detail in Stange *et al*.^46^.

#### Gross nitrification and NO_3_^−^ consumption rates

Gross nitrification and NO_3_^−^ consumption rates were determined using ^15^N pool dilution. Individual rates were determined for all time frames (0–1, 1–2, 2–4, 4–7 and 7–10 days) based on the approach first described by Kirkham and Bartholomew^47^ using the equations from Davidson *et al*.^48^. The equations (1–2) can be found in the Supplementary Information.

### Quantitative polymerase chain reaction (qPCR) analyses

#### Nucleic acid extraction

For quantitative polymerase chain reaction (qPCR) analyses nucleic acids (RNA and DNA) were extracted from frozen (−80 °C) soil sub samples taken at each time point of sampling (0, 1, 2, 4, 7, 10 days) of experiment 1. Total RNA and DNA were extracted simultaneously from the moist equivalent of 2 g dry soil using the RNA PowerSoil Total RNA Isolation Kit in combination with the RNA PowerSoil DNA Elution Accessory Kit according to the manufacturer protocol (MO BIO Laboratories, Carlsbad, CA, USA). Concentration and quality of RNA and DNA isolates were determined spectrophotometrically (NanoDrop 1000, Thermo Scientific, Waltham, MA, USA), fluorometrically (Qubit 2.0 Fluorometer, Life Technologies, Carlsbad, CA, USA), and by gel electrophoresis (Experion Automated Electrophoresis Station, Bio-Rad Laboratories, Hercules, CA, USA).

#### Reverse transcription

Digestion of residual DNA in RNA extracts was performed using the Ambion TURBO DNA-free Kit (Life Technologies, Carlsbad, CA, USA) according to the instructions given by the manufacturer. Successful DNA removal was confirmed by PCR using primers 27F (5′-AGAGTTTGATCMTGGCTCAG-3′)^49^ and PC5 (5′-TACCTTGTTACGACTT-3′)^50^ with the following conditions: hot start at 70 °C, 5 min at 95 °C, 35 cycles with 1 min at 95 °C, 1 min at 44 °C and 3 min at 72 °C followed by a final elongation step of 10 min at 72 °C. If no PCR products were obtained, RNA extracts were used for cDNA synthesis via reverse transcription. Reverse transcription of DNA-free RNA extracts was performed with random primers (Life Technologies, Carlsbad, CA, USA) using the SuperScript III Reverse Transcriptase (Life Technologies, Carlsbad, CA, USA) according to the manufacturer’ s protocol.

#### Nucleic acid extraction efficiencies

To allow the determination of DNA and RNA extraction efficiencies of each sample we added prior to extraction 4 × 10^10^ DNA and 7 × 10^10^ RNA copies of a linearized plasmid carrying a fragment of a cassava mosaic virus (accession number: AJ427910) as internal standard. This fragment is not thought to naturally occur in our soil and biochar and has been used as internal standard to monitor DNA recovery before^51^. In order to determine DNA and RNA extraction efficiencies the number of internal standard copies in the final DNA and cDNA extracts was quantified using qPCR. Extraction efficiencies were calculated according to equation (3) in the Supplementary Information.

#### Quantitative polymerase chain reaction (qPCR)

The quantification of internal standard copies (DNA and cDNA) and functional marker genes (DNA) and transcripts (cDNA) (*napA, narG, nirK, nirS*, typical *nosZ,* and atypical *nosZ*) was carried out by qPCR using the iCycler iQ Real-Time PCR Detection System and the iQ 5 Optical System software (Bio-Rad Laboratories, Hercules, CA, USA). qPCRs were setup in 96 well plates with a reaction volume of 10 μl. Reaction mixtures consisted of SsoAdvanced Universal SYBR Green Supermix or IQ SYBR Green Supermix (Bio-Rad Laboratories, Hercules, CA. USA), gene-specific primers and template DNA or cDNA. As standards, dilution series with plasmids containing fragments of the target genes were used. In all qPCRs, standards, samples and negative controls were analysed in triplicates and amplicon specificity was confirmed by performing melt curve analyses and 2% agarose gels. To account for potential differences in extraction efficiency, gene and transcript copy numbers were divided by the corresponding average control and biochar extraction efficiencies. Details about the qPCRs are listed in Tables S1 and S2 in the Supplementary Information.

### Gas analyses

#### Determination of concentrations and isotopic signatures of N_2_O in experiment 1

N_2_O concentrations in the 20 ml septum-capped glass vials sampled from the gas enrichment containers were measured using a gas chromatograph (GC) equipped with an electron capture detector (^63^Ni-ECD) (5890 Series II, HP, Palo Alto, CA, USA). The GC setup and configuration has been described in detail in Loftfield *et al*.^52^. The precision of N_2_O determination, expressed as the coefficient of variation for repeated standard gas quantifications, was below 3%.

The abundance of ^15^N-substituted isotopologues of N_2_O i.e. (^14^N^15^N^16^O + ^15^N^14^N^16^O) and ^15^N^15^N^16^O was quantified in the gas samples collected in the 120 ml septum-capped glass vials from the same gas enrichment containers. Isotopic signatures of N_2_O molecules were determined by analysing m/z 44, 45 and 46 using an isotope ratio mass spectrometer (IRMS, Delta^Plus^ XP IRMS, Thermo Finnigan, Waltham, MA, USA). The IRMS was connected to a pre-concentration device, equipped with a chemical CO_2_-trap and an automated cryo-trap (PreCon, Thermo Finnigan, Waltham, MA, USA), and a Gasbench II (Thermo Finnigan, Waltham, MA, USA). A CTC PAL autosampler (CTC Analytics AG, Zwingen, Switzerland) was used to collect gas from the sample vials by flushing the vials with a continuous flow of He carrier gas (25 ml/min). The precision for ^45^R and ^46^R determination, expressed as the coefficient of variation for repeated standard gas quantifications at different N_2_O concentrations was below 1.6% and 1.1%, respectively.

#### Determination of isotopic signatures of N_2_O and N_2_ in experiment 2 and 3

In experiment 2 and 3 abundances of ^15^N substituted isotopologues of N_2_ (^15^N^14^N, ^15^N^15^N) and N_2_O ([^14^N^15^N^16^O + ^15^N^14^N^16^O] and ^15^N^15^N^16^O) were determined in the gas samples collected in the 12 ml screw-cap exetainers during gas enrichment. Isotopologues of N_2_O and N_2_ were analysed using a modified GasBench II preparation system coupled to a MAT 253 isotope ratio mass spectrometer (Thermo Finnigan, Waltham, MA, USA) as described in Lewicka-Szczebak *et al*.^53^. In this setup N_2_O is converted to N_2_ prior to analysis enabling the simultaneous quantification of ^29^R (^29^N_2_/^28^N_2_) and ^30^R (^30^N_2_/^29^N_2_) of N_2_, N_2_O + N_2_ and N_2_O. The precision of ^29^R and ^30^R determination of this setup is below 0.15% and 1%, respectively^53^.

### N_2_O source partitioning and rates of NO_3_^−^-derived N_2_O and N_2_

Apart from slight modifications at some steps the general analysis procedure was performed according to Buchen *et al*.^54^. We used the non-equilibrium approach^55^,^56^ to calculate the ^15^N enrichment of the N_2_O-producing NO_3_^−^ pool (a_*p*_) and the fractions of pool-derived (NO_3_^−^) N_2_O and N_2_ (f_*p*_) using the equations described in Spott *et al*.^57^. Details about the used equations (4–9) can be found in the Supplementary Information.

Concentrations of NO_3_^−^-derived N_2_O and N_2_ (*c*_*p*_) were determined by multiplication of f_*p*_ values with the headspace concentration of total N_2_O and N_2_ (equation (10) in the Supplementary Information). *c*_*p*_ values were corrected for dilution effects (cc_*p*_) when needed and subsequently used to calculate NO_3_^−^-derived N_2_O and N_2_ emission rates (*ER*_*p*_) using equations (11–13) and (14–15) respectively, as described in the Supplementary Information. NO_3_^−^-derived N_2_O and N_2_ soil entrapment rates (*SER*_*p*_) and total production rates (*TPR*_*p*_) were determined based on the soil entrapment/emission ratio (*SE*_*p*_/*E*_*p*_) using equations (16–18) in the Supplementary Information.

The contribution of N_2_O from the labelled NO_3_^−^ pool to total soil-derived N_2_O emissions (f_*nitrate*_) was determined using equation (19), as described in the Supplementary Information.

### Statistical analysis

The effects of biochar addition and time (main effects) as well as their interaction (biochar*time) on NO_3_^−^-derived N_2_O and N_2_ emission rates, NO_3_^−^-derived N_2_O/(N_2_O + N_2_) emission ratios and the numbers of gene and transcript copies were evaluated using two-way ANOVA. Two-way ANOVAs were carried out in SAS (SAS 9.2, SAS Institute, Cary, NC, USA) using PROC MIXED. The model is given by:


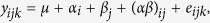


where y_*ijk*_ is the *k *th replicate at the *i *th biochar treatment at the *j *th time point. *μ* is the total effect, *a*_*i*_ is the main effect of the *i* th biochar treatment (control or biochar), *β*_*j*_ is the main effect of the *j* th time point (days 0, 1, 2, 4, 7, 10) and (*αβ*)_*ij*_ is the interaction of the *i* th biochar treatment and the *j* th time point. e_*ijk*_ is the error of y_*ijk*_.

In case it increased the model fit, group specific error variances were considered. Selection of the best fitting model was based on the Akaike Information Criterion (AIC). If necessary, a logarithmic transformation was used to reach normally distributed residuals with homogeneous variances. For data presentation log transformed means were back transformed. The corresponding standard errors were back transformed using the delta method. When significant interaction effects were determined simple mean comparisons were performed using the SLICE statement in PROC MIXED.

Temporal averages of the sources of N_2_O emissions, individual and mean nitrogen transformation rates, cumulative NO_3_^−^-derived N_2_O and N_2_ emissions, as well as NO_3_^−^-derived N_2_O and N_2_ emission, soil entrapment and total production rates determined only at day 2 in experiment 3 were analysed for differences in control and biochar microcosms using t-tests. In addition, t-tests were applied to evaluate differences between NO_3_^−^-derived N_2_O and N_2_ emission and soil entrapment rates within treatments (control or biochar). T-tests were carried out in SAS using PROC TTEST.

## Results

### Gross nitrification and NO_3_^−^ consumption rates

Individual gross nitrification rates (experiment 1; days 0–1, 1–2, 2–4, 4–7, 7–10) ranged from 0.07 ± 0.02 to 0.28 ± 0.03 mg N kg^−1^ dry soil h^−1^ (Table 2). Comparison of control and biochar microcosms indicated slightly higher mean nitrification rates in control compared to biochar-amended microcosms. However, individual gross nitrification rates were only significantly higher in control microcosms between days 2–4 and between days 7–10. For all other time periods, rates were either equal (days 4–7) or higher in biochar compared to control microcosms (days 0–1 and 1–2).

Individual gross NO_3_^−^ consumption rates (experiment 1; days 0–1, 1–2, 2–4, 4–7, and 7–10) ranged from 0.07 ± 0.02 to 0.61 ± 0.04 mg N kg^−1^ dry soil h^−1^ (Table 2). Mean NO_3_^−^ consumption rates were higher in biochar-amended than in control microcosms and also individual NO_3_^−^ consumption rates were higher in biochar microcosms for 4 out of 5 time periods. Significantly higher individual rates in biochar microcosms were determined between day 0 and 1 and between day 7 and 10.

In control microcosms mean nitrification and NO_3_^−^ consumption rates were very similar, 0.19 ± 0.02 and 0.20 ± 0.05 mg N kg^−1^ dry soil h^−1^, respectively. In biochar-amended microcosms, however, the mean NO_3_^−^ consumption rate was significantly (*p* = 0.017) higher (0.3 ± 0.05 mg N kg^−1^ dry soil h^−1^) than the mean nitrification rate (0.16 ± 0.02 mg N kg^−1^ dry soil^−1^).

### Sources of N_2_O emissions

N_2_O source partitioning (experiment 1) indicated that the predominant source of N_2_O emissions was the ^15^N-labelled NO_3_^−^ pool. On average 100.9 ± 1.7% (control) and 100.1 ± 2.3% (biochar-amended microcosms) of total soil-derived N_2_O emissions originated from the ^15^N-labelled NO_3_^−^ pool. Statistically significant differences between control and biochar microcosms were not detected. The contribution of NO_3_^−^-derived N_2_O to total soil-derived N_2_O emissions over time is shown in Figure S1 in the Supplementary Information.

### NO_3_^−^-derived N_2_O and N_2_ emissions

**Figure 1:**
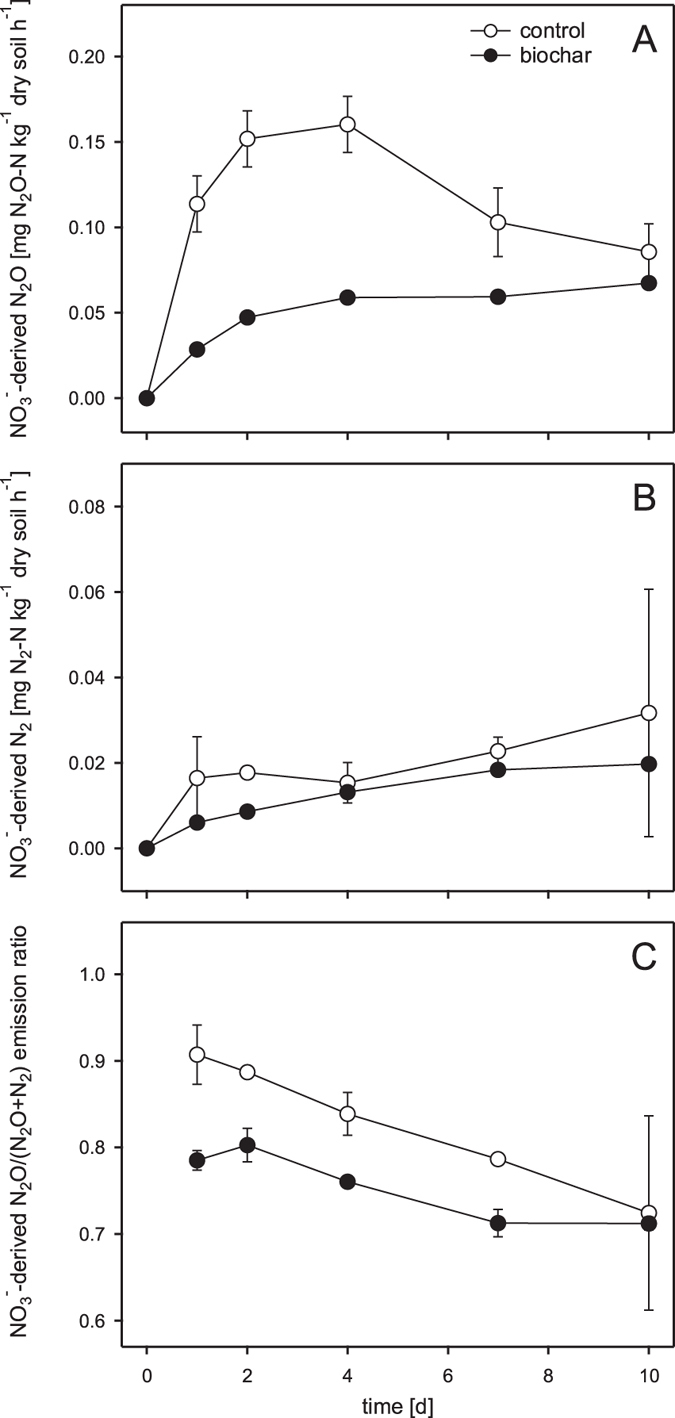
NO_3_^−^-derived N_2_O emission rates (A), NO_3_^−^-derived N_2_ emission rates (B), and the NO_3_^−^-derived N_2_O/(N_2_O + N_2_) emission ratio (C) in control (white circles) and biochar (black circles) microcosms over time (experiments 1 and 2). Data points and error bars represent means and standard errors (n = 3), respectively.

Time courses of NO_3_^−^-derived N_2_O emission rates (experiments 1) differed strongly in control and biochar-amended microcosms (Fig. 1A). In general, emission rates were much higher in control compared to biochar microcosms. Furthermore, the addition of biochar also changed the temporal dynamics of NO_3_^−^-derived N_2_O emission rates. In control microcosms the highest rates were quantified after 4 days of incubation (0.160 mg N_2_O-N kg^−1^ dry soil h^−1^). In the presence of biochar, highest NO_3_^−^-derived N_2_O emission rates were determined at day 10 (0.067 mg N_2_O-N kg^−1^ dry soil h^−1^). Between day 1 and 10 emission rates were always higher in control microcosms. Two-way ANOVA revealed that biochar amendment and time as well as their interaction (biochar*time) had a significant effect (Table 3). Significantly higher emission rates in control compared to biochar microcosms were determined at day 1, 2 and 4 (*p* < 0.001 for each time point). Cumulative (day 0–10) NO_3_^−^-derived N_2_O emissions were significantly (p = 0.004) higher in control (27.2 ± 1.9 mg N_2_O-N kg^−1^ dry soil) than in biochar (12.6 ± 0.2 mg N_2_O-N kg^−1^ dry soil) microcosms resulting in biochar-induced N_2_O emission mitigation of 54 ± 16%.

NO_3_^−^-derived N_2_ emission rates (experiment 2) were substantially lower than the corresponding NO_3_^−^-derived N_2_O emission rates and showed only minor differences between control and biochar-amended microcosms (Fig. 1B). Highest rates, with values of 0.03 mg N_2_-N kg^−1^ dry soil h^−1^ in control and 0.02 mg N_2_-N kg^−1^ dry soil h^−1^ in biochar microcosms, were observed at day 10. Between day 1 and 10, emission rates were slightly higher in control microcosms. According to the two-way ANOVA, NO_3_^−^-derived N_2_ emission rates were not significantly affected by biochar addition, time or their interaction (Table 3). Cumulative (day 0–10) NO_3_^−^-derived N_2_ emissions were slightly (control: 5.3 ± 1.4 mg N_2_-N kg^−1^ dry soil, biochar: 3.3 ± 0.2 mg N_2_-N kg^−1^ dry soil), but not statistically significantly higher in control microcosms.

The NO_3_^−^-derived N_2_O/(N_2_O + N_2_) emission ratio (experiment 2) ranged from 0.71 to 0.91 (Fig. 1C). The ratio was always lower in biochar microcosms at each time point of sampling from day 1 to day 10. Nonetheless, two-way ANOVA revealed that in contrast to time, which had a significant effect on the N_2_O/(N_2_O + N_2_) emission ratio, the effect of biochar addition as well as the interaction of time and biochar did not significantly affect the N_2_O/(N_2_O + N_2_) ratio (Table 3).

### Gene and transcript copies of functional denitrification genes

**Figure 2:**
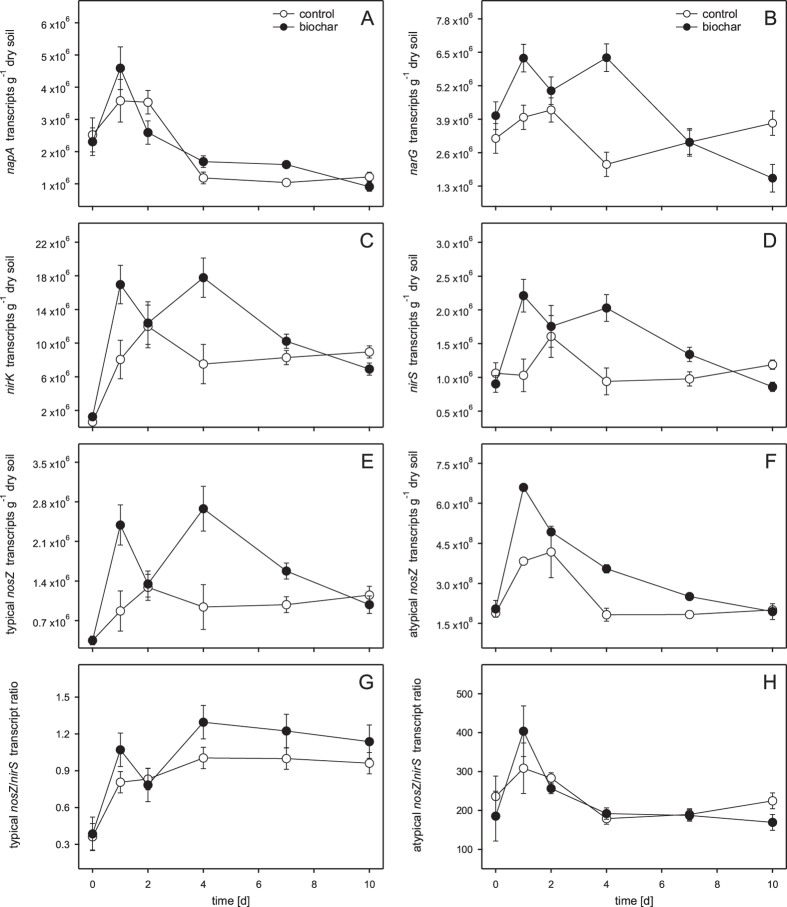
Transcript copy numbers of functional marker genes of denitrification in control (white circles) and biochar (black circles) microcosms over time (experiment 1). The different panels show: *napA* (**A**), *narG* (**B**), *nirK* (**C**), *nirS* (**D**), typical *nosZ* (**E**), and atypical *nosZ* (**F**). Panels G and H show the typical *nosZ/nirS* and the atypical *nosZ/nirS* transcript ratio, respectively. Data points and error bars represent means and standard errors (n = 3), respectively.

*napA, narG, nirK, nirS*, typical *nosZ* and atypical *nosZ* transcript copy numbers (experiment 1) were strongly affected by biochar addition and changed as a function of time (Fig. 2). For *napA, narG* and *nirS* transcript copy numbers two-way ANOVA revealed a significant interaction (biochar*time), indicating that biochar effects were correlated to the time of sampling (Table 3).

*napA* transcript levels were relatively similar in control and biochar microcosms and ranged from 9.1 × 10^5^ to 4.6 × 10^6^ transcripts g^−1^ dry soil (Fig. 2A). Significantly more *napA* transcripts in biochar microcosms were quantified at day 7 (*p* = 0.022). *narG* ranged from 1.6 × 10^6^ to 6.3 × 10^6^ transcripts g^−1^ dry soil and showed pronounced differences between control and biochar microcosms with higher values in biochar microcosms at day 1, 2 and 4, and lower transcript levels in the biochar setups at day 10 (Fig. 2B). Significantly higher *narG* transcript copy numbers in biochar microcosms were quantified at day 4 (p < 0.001) and significantly lower values were determined at day 10 (*p* = 0.004). *nirS* transcript copy numbers ranged from 8.6 × 10^5^ to 2.2 × 10^6^ transcripts g^−1^ dry soil (Fig. 2D). Transcript levels were higher in biochar microcosms at day 1, 2, 4, and 7 with significantly higher values at day 1 (*p* = 0.026) and 4 (*p* = 0.018). Significantly lower values were quantified after 10 days of incubation (*p* = 0.027).

Transcript copy numbers of *nirK*, typical *nosZ* and atypical *nosZ* were significantly affected by both main effects, biochar addition and time (Table 3). Transcript copy numbers of *nirK,* typical *nosZ and* atypical *nosZ* ranged from 6.3 × 10^5^ to 1.8 × 10^7^, 3.5 × 10^5^ to 2.7 × 10^6^, and 3.3 × 10^6^ to 6.6 × 10^8^ transcript copies g^−1^ dry soil, respectively. All three were higher in biochar microcosms at days 1, 2, 4 and 7 (Fig. 2C). According to two-way ANOVA *nirK*, typical *nosZ* and atypical *nosZ* transcript copy numbers were significantly higher in biochar microcosms compared to the control microcosms (Table 3).

While some of the transcript copy ratios were affected by biochar addition and time (typical *nosZ/nirS* and atypical *nosZ/nirS*; Fig. 2G,H), others showed similar values in control and biochar microcosms and hardly changed over time (typical *nosZ/nirK* and atypical *nosZ/nirK*; time course data not shown). Time courses for the typical *nosZ/nirS* transcript ratio ranged from values of 0.36 to 1.29 (Fig. 2G). The typical *nosZ/nirS* transcript ratio was higher in the biochar microcosms at days 1, 4, 7, and 10 and two-way ANOVA revealed significant time (*p* < 0.001) and biochar effects (*p* = 0.031) (Table 3). The atypical *nosZ/nirS* transcript ratio ranged from values of 179.1 to 404.2 and was hardly affected by biochar addition. In contrast to the typical *nosZ/nirS* transcript ratio, time was the only significant effect on the atypical *nosZ/nirS* transcript ratio (Table 3). According to two-way ANOVA, no significant effects (biochar, time, biochar*time) could be determined for typical *nosZ/nirK* and atypical *nosZ/nirK* transcript ratios (Table 3).

Gene copy numbers of *napA, narG, nirK, nirS*, typical *nosZ* and atypical *nosZ* showed similar dynamics as the corresponding transcripts (Figure S2). However, differences between control and biochar microcosms were less prominent. Two-way ANOVA revealed a significant biochar effect on *napA* gene abundance, a significant biochar*time effect on typical *nosZ*, and significant time and biochar*time effects on *nirS* gene copy numbers (Table S3).

### Soil entrapment of NO_3_^−^-derived N_2_O and N_2_

**Figure 3:**
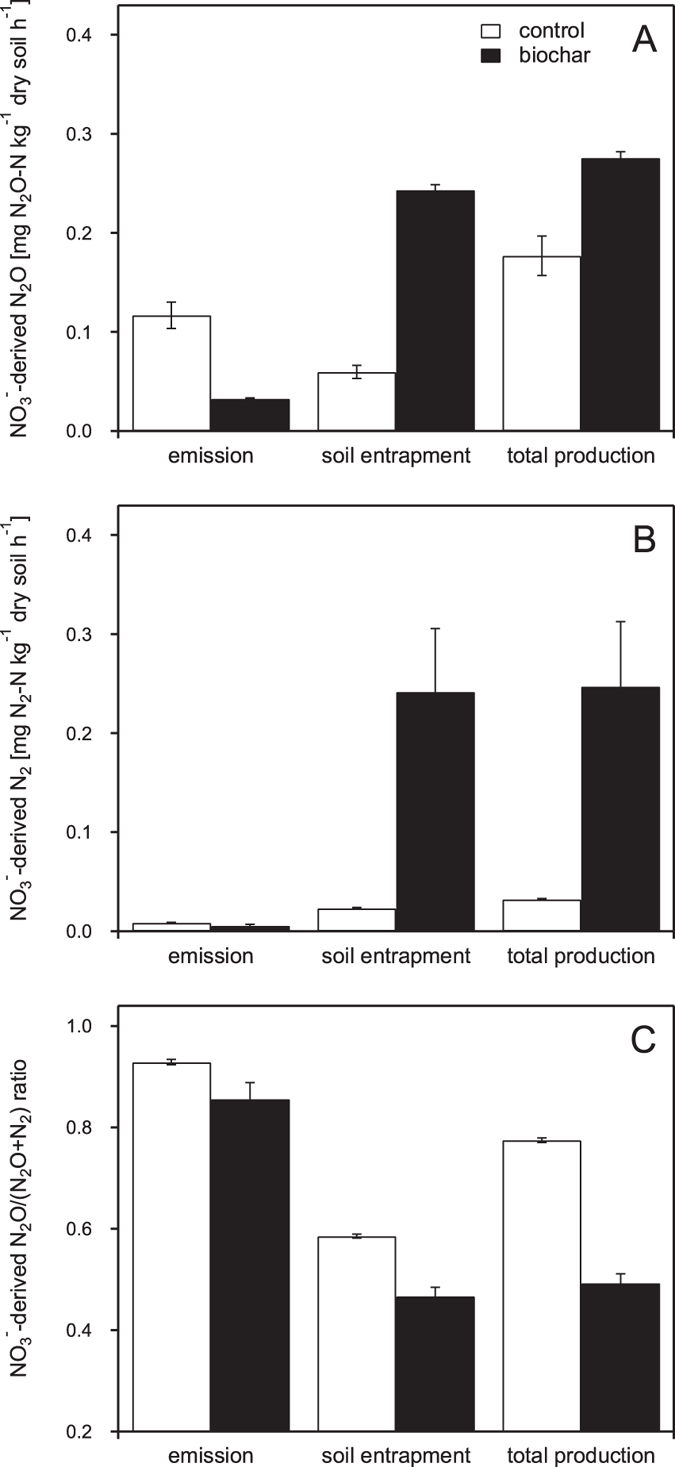
Emission, soil entrapment and total production rates of NO_3_^−^-derived N_2_O (A) and N_2_ (B) in control (white bars) and biochar (black bars) microcosms at day 2 (experiment 3). Panel C shows the N_2_O/(N_2_O + N_2_) product ratio derived from (A) and (B). Bars and error indicators represent means and standard errors (n = 3), respectively.

Quantification of NO_3_^−^-derived N_2_O and N_2_ remaining in the soil at day 2 (experiment 3) revealed that the extent of soil gas content significantly differed in control and biochar-amended microcosms and strongly depended on the type of nitrogen gas (N_2_O, N_2_) (Fig. 3).

In control microcosms soil entrapment/emission ratios (*SE*_*p*_/*E*_*p*_) for NO_3_^−^-derived N_2_O and N_2_ were 0.5 and 2.7, respectively. Accordingly, emission rates were significantly higher than soil entrapment rates for NO_3_^−^-derived N_2_O (p = 0.018) and significantly lower for NO_3_-derived N_2_ (p < 0.001) (Fig. 3A,B). In biochar microcosms the soil entrapment/emission ratios (*SE*_*p*_/*E*_*p*_) reached values of 7.5 (NO_3_^−^-derived N_2_O) and 42.9 (NO_3_^−^-derived N_2_) resulting in significantly higher soil entrapment than emission rates for both NO_3_^−^-derived N_2_O (p < 0.001) and N_2_ (p = 0.021) (Fig. 3A,B). Comparison of NO_3_^−^-derived N_2_O emission, soil entrapment, and total production rates in control and biochar microcosms revealed that while biochar addition significantly decreased the emission rate (*p* = 0.003), soil entrapment (*p* < 0.001) and total production rates (*p* = 0.009) were significantly higher in biochar-amended compared to control microcosms (Fig. 3A). Similar results were found for NO_3_^−^-derived N_2_. Although biochar addition did not significantly affect the NO_3_^−^-derived N_2_ emission rate, it significantly increased NO_3_^−^-derived N_2_ soil entrapment (*p* = 0.027) and total production (*p* = 0.030) rates (Fig. 3B).

Independent of biochar addition, NO_3_^−^-derived N_2_O/(N_2_O + N_2_) ratios were significantly higher for emitted than for entrapped gases (control: *p* < 0.001, biochar: *p* < 0.001) (Fig. 3C). Comparison of NO_3_^−^-derived N_2_O/(N_2_O + N_2_) ratios in control and biochar microcosms revealed a significant biochar effect. While soil biochar amendment did not significantly change the ratio of NO_3_^−^-derived N_2_O and N_2_ that was emitted, it significantly decreased the ratio of NO_3_^−^-derived N_2_O and N_2_ that remained in the soil (*p* = 0.003) and the ratio of total NO_3_^−^-derived N_2_O and N_2_ that was produced (*p* < 0.001) (Fig. 3C).

### Nitrogen balance

Nitrogen balances for the first 2 days of incubation (experiments 1, 2, and 3) were estimated based on the initial (day 0) and residual (day 2) NO_3_^−^ and NH_4_^+^ contents and the cumulative (day 0–2) NO_3_^−^-derived N_2_O and N_2_ losses (Table 4). Scenario I considered only emitted cumulative N_2_O and N_2_. Scenario II considered emitted and soil entrapped cumulative N_2_O and N_2_. In scenario I, 66.9 and 94.5% of the lost N from the mineral nitrogen pool (NO_3_^−^ and NH_4_^+^) remained unaccounted in control and biochar-amended microcosms, respectively. Cumulative emissions of NO_3_^−^-derived N_2_O and N_2_ accounted for only 33.1 (control) and 5.5% (biochar), respectively. In scenario II, which considered emitted and soil entrapped gases, cumulative NO_3_^−^-derived N_2_O and N_2_ accounted for 58.6 (control) and 79.1% (biochar) of the lost N. Thus, only 41.4 and 20.9% of the lost N remained unaccounted in control and biochar microcosms, respectively.

## Discussion

In fertilized soil systems, highest N_2_O emission rates frequently occur after heavy rainfall when water-filled pore spaces (WFPS) are high (i.e. O_2_ availability in the soil is low) and anaerobic nitrogen transformation processes such as denitrification are prevailing^21^,^22^. In order to simulate such situations, we set up a short-term soil microcosm experiment with a WFPS of 90% to which we added a NH_4_^15^NO_3_ solution to prevent NH_4_^+^ and NO_3_^−^ limitation for microbial nitrogen transformation processes.

N_2_O source partitioning revealed that soil biochar amendment did not significantly affect the sources of N_2_O. Independent of biochar addition, N_2_O emissions solely originated from the NO_3_^−^ pool suggesting that denitrification was the main N_2_O-producing pathway. This finding is in good agreement with other studies which were carried out under similar environmental conditions^58^,^59^,^60^.

Individual gross NO_3_^−^ consumption rates were higher in biochar microcosms for 4 out of 5 time periods when compared to the control microcosms. These results are in line with the findings of Prommer *et al*.^61^ and Hu *et al*.^62^ who also documented a biochar-induced increase in NO_3_^−^ consumption. According to the ^15^N pool dilution approach used in this study, gross NO_3_^−^ consumption covers microbial NO_3_^−^ assimilation, dissimilatory nitrate reduction to ammonium (DNRA) and denitrification^48^. However, the environmental conditions in the soil microcosms suggest that the majority of NO_3_^−^ was consumed by microbial denitrification. This is in contrast to other studies which assumed that a decrease in the denitrification rate would provide a potential explanation for decreased N_2_O emissions from biochar-amended soil^38^,^39^. However, Xu *et al*.^15^ and Jones *et al*.^63^ quantified higher denitrification enzyme activities (DEA) in the presence of biochar. Also Obia *et al*.^40^ quantified higher denitrification rates in biochar-amended soil slurries.

Although gross NO_3_^−^ consumption rates were higher, NO_3_^−^-derived N_2_O emission rates were significantly lower in biochar-amended microcosms. Furthermore, the calculation of cumulative NO_3_^−^-derived N_2_O emissions showed that soil biochar amendment significantly decreased N_2_O emissions by 54%. These results confirmed what has been shown in several lab and field studies before^11^,^64^,^65^,^66^,^67^,^68^,^69^, that biochar amendment can mitigate soil N_2_O emission. In addition to N_2_O emission reduction, Cayuela and colleagues showed that soil biochar amendment substantially decreased the N_2_O/(N_2_O + N_2_) emission ratio^64^. Similar effects were also found in the soil slurry experiments by Obia *et al*.^40^ as well as in this study. Although two-way ANOVA revealed no significant biochar, time, or interaction effects, biochar addition decreased the NO_3_^−^-derived N_2_O/(N_2_O + N_2_) emission ratio at all sampling points. In contrast to Obia *et al*.^40^ who reported an increase in N_2_ emissions following biochar addition, NO_3_^−^-derived N_2_ emission rates and cumulative NO_3_^−^-derived N_2_ emissions were almost equal in control and biochar microcosms in our study. Thus, according to the NO_3_^−^-derived N_2_O and N_2_ emission data, biochar addition decreased total denitrification-related nitrogen loss (NO_3_^−^-derived N_2_O + N_2_).

Several studies showed that in flooded and wet soils significant quantities of N_2_O and N_2_ can remain entrapped in the soil matrix^45^,^70^,^71^. As diffusion coefficients are significantly lower (approximately 4 orders of magnitude) in water compared to air, diffusion of microbially-released N_2_O and N_2_ from the site of production to the soil surface takes considerably longer in soils with elevated water-filled pore space compared to well-aerated soils^34^,^35^. Furthermore, N_2_O and N_2_ emission can be suppressed when N_2_O and N_2_ accumulate in gas bubbles entrapped within the water-saturated pore network^34^,^72^. For flooded soils it has been reported that gas sampling after physically disturbing the soil (e.g. by shaking the soil microcosm), resulted in up to 12 times more N_2_O and N_2_ than could be quantified in the headspace of undisturbed microcosms^45^,^70^,^71^. We also showed that physical destruction of the soil matrix by shaking and subsequent gas sampling led to significantly higher quantities of NO_3_^−^-derived N_2_O and N_2_ in the microcosm headspace. Interestingly, the nitrogen gas entrapment in the soil (both NO_3_^−^-derived N_2_O and N_2_) was significantly higher in the presence of biochar. It remains to be shown if this increase in soil gas entrapment is a direct biochar effect e.g. by sorption of N_2_O and N_2_ onto biochar particles^41^,^42^ or related to biochar-induced alterations of the soil pore structure and soil aggregation that resulted in changes of the soil hydraulic properties^73^,^74^.

The high relevance of N_2_O and N_2_ soil entrapment was also supported by nitrogen balance calculations. When only gas emission data from undisturbed microcosms was taken into account, a relatively high fraction of nitrogen could not be accounted for in the nitrogen balance calculations. However, this fraction decreased substantially when headspace gas and soil entrapped nitrogen gases (i.e. total production) were both considered. This indicates that NO_3_^−^-derived N_2_O and N_2_ represented the major sinks of mineral nitrogen, even though a substantial fraction was not accounted for by headspace sampling from undisturbed microcosms. Due to the high entrapment rate and the higher NO_3_^−^ consumption rates, biochar significantly increased total production rates of NO_3_^−^-derived N_2_O and N_2_ (sum of entrapped and emitted gases). Additionally, NO_3_^−^-derived N_2_O/(N_2_O + N_2_) ratios calculated based on total production rates were significantly lower in biochar-amended microcosms. These findings suggest that when total production rates and not only emission rates were considered, biochar actually increased denitrification-related nitrogen losses. However, as indicated by the lower product ratio, biochar addition significantly decreased the relative contribution of N_2_O to the total nitrogen gas loss.

Although in the present study biochar slightly increased *napA, narG, nirK, nirS*, typical *nosZ* and atypical *nosZ* gene copy numbers, the observed differences were mostly not statistically significant. This could be explained by the short incubation time and the fact that no carbon source was added. Thus no significant changes in the abundance of the denitrifying microbial community were observed in response to soil biochar amendment. No significant effect of biochar on gene copy numbers of functional denitrification genes has also been reported in other studies^75^,^76^. In contrast to our results on gene copy numbers, soil biochar amendment significantly increased *nirK*, typical *nosZ*, and atypical *nosZ* transcript copy numbers. Furthermore, *narG* and *nirS* transcript levels were elevated in biochar microcosms at most time points with significantly higher values only at certain dates. Higher numbers of nitrate, nitrite and nitrous oxide reductase transcripts suggest a higher denitrification activity in biochar-amended microcosms. This is in accordance with the higher gross NO_3_^−^ consumption rates. Interestingly biochar addition most significantly increased typical and atypical *nosZ* transcript copy numbers indicating a biochar-induced promotion of N_2_O-reducing microorganisms. In consequence, the typical *nosZ/nirS* transcript ratio, i.e. the number of expressed nitrous oxide reductase genes per expressed nitrite reductase genes, was significantly higher in biochar microcosms. These results are in good agreement with other studies in which functional denitrification genes^14^,^16^,^77^ and their mRNA^15^,^16^ have been quantified and suggest that biochar addition enhances microbial N_2_O reduction. As reported in several studies nitrous oxide reductases are highly pH sensitive and are impaired due to post-transcriptional effects at pH values below 6.1^25^,^78^,^79^. Obia *et al*.^40^ showed in soil slurry experiments with acidic soil that the addition of alkaline biochar significantly increased the pH of the slurries and decreased the N_2_O/(N_2_O + N_2_) product ratio. Based on their findings the authors concluded that due to the higher pH in biochar-amended slurries impairment of nitrous oxide reductases was less prominent, which resulted in a higher N_2_O reduction activity^40^. In the present study, however, we used a slightly alkaline soil. Measured pH values never dropped below 7.6 at any sampling time point of our microcosm experiment (data not shown). Although we cannot exclude that pH values were lower at specific microsites of the soil, for example due to the oxidation of NH_4_^+^ during nitrification^80^ it is unlikely that pH values dropped below 6.1 frequently. Therefore, we think that pH-driven impairment of nitrous oxide reductases did not represent a major controlling factor for N_2_O reduction activity in the present study.

In the present soil microcosm study, which was conducted with a slightly alkaline sandy clay loam soil and a wood-derived biochar, the observed biochar-induced N_2_O emission mitigation and lower N_2_O/(N_2_O + N_2_) ratio can potentially be explained by an interrelation of soil physical and microbiological parameters. Our results suggest that under the environmental conditions (N fertilization, high WFPS) of the present study, N_2_O soil entrapment is the main mechanism causing N_2_O emission mitigation. In biochar-amended microcosms we quantified 4.1 times more soil matrix entrapped NO_3_^−^-derived N_2_O compared to the control microcosms. In addition, we determined an overall decrease of the N_2_O/(N_2_O + N_2_) ratio which was correlated to significantly increased typical and atypical *nosZ* transcript copy numbers and an increased typical *nosZ/nirS* transcript ratio, suggesting that soil biochar amendment enhanced N_2_O reduction activity of “classical” denitrifiers and atypical N_2_O reducers. Recent studies revealed that about half of the so far detected atypical *nosZ* gene carrying N_2_O reducers lack other functional denitrification genes and thus depend on the supply of N_2_O by other organisms^32^,^33^. These microorganisms as well as typical *nosZ* containing N_2_O reducers that specialized on N_2_O reduction^81^ might benefit from the entrapped N_2_O released by denitrifiers not capable of N_2_O reduction^26^, and further reduce it to N_2_. Support for this hypothesis comes from a recent sequencing study that revealed a biochar-induced increase in relative sequence abundance of microbial species carrying atypical *nosZ* genes that lack other denitrification genes and classical denitrifier species efficiently performing complete denitrification^82^. According to Clough *et al*.^34^ the potential for microbial reduction of N_2_O to N_2_ increases when N_2_O remains entrapped in the soil pore space. It is conceivable that an increase in N_2_O availability as consequence of the increased retention time of N_2_O in water-filled pores, stimulated microbial N_2_O reduction and thus decreased the N_2_O/(N_2_O + N_2_) ratio. We cannot exclude, however, that abiotic N_2_O reduction reactions with redox active organic compounds or organo-mineral phases high in Fe also contributed to the lower N_2_O/(N_2_O + N_2_) ratio in soil microcosms amended with biochar^42^.

In conclusion, this study confirmed that biochar amendment can significantly decrease N_2_O emissions from a slightly alkaline sandy clay loam soil under denitrifying conditions. Interestingly, N_2_O emission mitigation occurred although biochar addition stimulated denitrification gene expression, increased denitrification rates, and elevated total N_2_O and N_2_ production rates. Our data suggest that the lower N_2_O emissions from biochar-amended soil in the present study are caused by N_2_O entrapment in water-saturated soil pores and consequent stimulation of microbial N_2_O reduction by classical denitrifiers and atypical N_2_O reducers leading to a lower N_2_O/(N_2_O + N_2_) product ratio. Our findings emphasize the importance of considering soil entrapped nitrogen gases in biochar studies, especially under conditions of an elevated WFPS. However, the data shown here is based on a plant-free, short-term microcosm experiment with one specific sandy clay loam soil and a wood-derived biochar. The relevance of N_2_O entrapment for other soil-biochar combinations and the conditions under which any soil entrapped N_2_O might be emitted (e.g. extreme events such as drought or tillage) should be systematically evaluated in future lab and field studies.

## Additional Information

**How to cite this article:** Harter, J. *et al*. Gas entrapment and microbial N_2_O reduction reduce N_2_O emissions from a biochar-amended sandy clay loam soil. *Sci. Rep.* **6**, 39574; doi: 10.1038/srep39574 (2016).

**Publisher's note:** Springer Nature remains neutral with regard to jurisdictional claims in published maps and institutional affiliations.

## Supplementary Material

Supplementary Information

## Figures and Tables

**Table 1:** Properties of soil and biochar.

property	unit	soil	biochar
pH_H2O_		7.59	9.18
C	%	3.42	73.2
CaCO_3_	%	5.03	na
C_org_	%	2.81	73.1
N	%	0.34	0.64
C_org_/N	%	8.26	114
H	%	na	1.03
O	%	na	5.70
H:C		na	0.17
O:C		na	0.06
Ash	%	na	19.4
EC	μS/cm	na	782
SA	m^2^/g	na	231
Ca	%	1.67	4.90
Fe	%	0.56	0.27
K	%	0.20	0.84
Mg	%	0.30	0.33
B	mg/kg	na	36
Cd	mg/kg	<0.2	<0.2
Cr	mg/kg	9.3	10
Cu	mg/kg	6.4	16
Hg	mg/kg	na	<0.07
Mn	mg/kg	109	310
Mo	mg/kg	<0.1	na
Na	mg/kg	13	830
Ni	mg/kg	6	8
P	mg/kg	208	1400
Pb	mg/kg	8	<2
S	mg/kg	228	400
Si	mg/kg	119	22000
Zn	mg/kg	32	45
PAHs	mg/kg	na	6.70

C_org_: organic carbon EC: electrical conductivity, SA: surface area, PAHs: polycyclic aromatic hydrocarbons (sum of the EPA’s 16 priority pollutants), na: not analyzed.

**Table 2:** Gross nitrification and NO_3_^−^ consumption rates in control and biochar microcosms (experiment 1).

time period [days]	nitrification rate [mg N kg^−1^ dry soil h^−1^]			NO_3_^−^ consumption rate [mg N kg^−1^ dry soil h^−1^]		
	control	biochar	p-value	control	biochar	p-value
0–1	0.24 ± 0.04	0.28 ± 0.03	0.504	0.28 ± 0.03	0.61 ± 0.04	**0.004**
1–2	0.08 ± 0.02	0.18 ± 0.03	0.075	0.49 ± 0.18	0.42 ± 0.05	0.683
2–4	0.20 ± 0.01	0.07 ± 0.02	**0.013**	0.08 ± 0.03	0.14 ± 0.02	0.190
4–7	0.1 ± 0.02	0.1 ± 0.00	0.941	0.17 ± 0.03	0.17 ± 0.02	0.871
7–10	0.28 ± 0.02	0.17 ± 0.00	**0.002**	0.07 ± 0.02	0.16 ± 0.01	**0.008**
mean	0.19 ± 0.02	0.16 ± 0.02	0.403	0.20 ± 0.05	0.30 ± 0.05	0.183

Individual rates were determined for all time frames between the sampling dates. For individual rates values represent means ± standard errors (n = 3). Mean rates were calculated using the individual rates from all time frames. For mean rates values represent means ± standard errors (n = 15). p-values originate from t-tests (control vs. biochar).

**Table 3:** Results from two-way ANOVAs for gas emission, transcript, and transcript ratio data (experiments 1 and 2).

parameter	biochar		time		biochar * time	
	F	p	F	p	F	p
NO_3_^−^-derived N_2_O emission rate	83.17	**<0.001**	3.86	**0.042**	5.07	**0.020**
NO_3_^−^-derived N_2_ emission rate	1.50	0.318	3.96	0.358	0.77	0.683
NO_3_^−^-derived N_2_O/(N_2_O + N_2_) emission ratio	9.06	0.060	10.06	**0.035**	0.28	0.877
*napA* transcripts	0.02	0.895	14.39	**0.002**	5.47	**0.024**
*narG* transcripts	3.95	0.064	3.91	**0.016**	6.46	**0.002**
*nirK* transcripts	5.71	**0.044**	9.34	**0.005**	3.64	0.060
*nirS* transcripts	11.45	**0.004**	4.19	**0.043**	6.77	**0.013**
typical *nosZ* transcripts	18.21	**<0.001**	20.87	**<0.001**	3.8	0.051
atypical *nosZ* transcripts	16.25	**0.006**	42.96	**0.004**	2.14	0.266
typical *nosZ/nirS* transcript ratio	5.36	**0.031**	11.51	**<0.001**	0.7	0.632
atypical *nosZ/nirS* transcript ratio	0.04	0.844	8.99	**0.006**	0.92	0.520
typical *nosZ/nirK* transcript ratio	0.02	0.906	1.95	0.207	0.57	0.725
atypical *nosZ/nirK* transcript ratio	0.66	0.492	6.13	0.060	0.49	0.770

The table shows F-statistics and p-values for the main effects “biochar” and “time” and their interaction “biochar*time”. Significant effects indicated by p-values below 0.05 are shown in bold font.

**Table 4:** Nitrogen balance after 2 days of incubation (experiments 1, 2, and 3).

	parameter	scenario I		scenario II	
		control	biochar	control	biochar
I_NO3_	initial NO_3_^−^-N [mg/kg]	239.1 ± 0.5	223.2 ± 0.9	239.1 ± 0.5	223.2 ± 0.9
I_NH4_	initial NH_4_^+^-N [mg/kg]	140.3 ± 2.5	139.6 ± 1.3	140.3 ± 2.5	139.6 ± 1.3
R_NO3_	residual NO_3_^−^-N after 2d [mg/kg]	239.5 ± 11.1	209.7 ± 1.0	239.5 ± 11.1	209.7 ± 1.0
R_NH4_	residual NH_4_^+^-N after 2d [mg/kg]	124.3 ± 7.5	125.7 ± 0.8	124.3 ± 7.5	125.7 ± 0.8
L_NO3+NH4_	lost N from NO_3_^−^ + NH_4_^+^ after 2d [mg/kg]	15.6 ± 3.2	27.3 ± 1.7	15.6 ± 3.2	27.3 ± 1.7
N_N2O_	NO_3_^−^-derived N_2_O-N after 2d [mg/kg]	4.6 ± 0.6	1.3 ± 0.0	6.9 ± 0.9	10.6 ± 0.2
N_N2_	NO_3_^−^-derived N_2_-N after 2d [mg/kg]	0.6 ± 0.2	0.2 ± 0.0	2.2 ± 0.9	10.9 ± 0.5
N_U_	unaccounted N after 2d [mg/kg]	10.4 ± 4.0	25.8 ± 1.7	6.5 ± 5.0	5.8 ± 2.4
P_N2O_	proportion of NO_3_^−^-derived N_2_O-N of lost N after 2d [%]	29.2 ± 7.0	4.6 ± 0.3	44.2 ± 10.6	39.0 ± 2.5
P_N2_	proportion of NO_3_^−^-derived N_2_-N of lost N after 2d [%]	3.9 ± 1.7	0.9 ± 0.1	14.4 ± 6.3	40.1 ± 3.0
P_U_	proportion of unaccounted N of lost N after 2d [%]	66.8 ± 8.7	94.5 ± 0.4	41.4 ± 16.9	20.9 ± 5.6

Values represent means ± standard errors (n = 3).

**L**_**NO3+NH4**_ = (I_NO3_ + I_NH4_) − (R_NO3_ + R_NH4_); **N**_**U**_ = L_NO3 + NH4_ − N_N2O_ − N_N2_; **P**_**N2O**_ = N_N2O_/L_NO3 + NH4_; **P**_**N2**_ = N_N2_/L_NO3 + NH4_; **P**_**U**_ = 1 − P_N2O_ − P_N2_.

**Scenario I**: N_N2O_ and N_N2_ are cumulative emissions extrapolated from the emission rates (*ER*_*p*_) determined at day 0, 1 and 2.

**Scenario II**: N_N2O_ and N_N2_ are cumulative total productions extrapolated from the emission rates (*ER*_*p*_) determined at day 0, 1 and 2, multiplied with the soil entrapment/emission ratio (*SE*_*p*_/*E*_*p*_) determined at day 2.
