# Supplementary material for: Gas entrapment and microbial N2O reduction reduce N2O emissions from a biochar-amended sandy clay loam soil
Source: Sci Rep. 2016 Dec 23;6:39574. doi: 10.1038/srep39574 (PMC5180216; doi:10.1038/srep39574)
Supplement: Supplementary Information [file srep39574-s1.pdf]

Gas entrapment and microbial N_2O reduction reduce N_2O emissions from a biochar-amended sandy clay loam soil

Johannes Harter [a], Ivan Guzman-Bustamante [b], Stefanie Kuehfuss [c], Reiner Ruser [b], Reinhard Well [d], Oliver Spott [e], Andreas Kappler [a] and Sebastian Behrens [a,f,g*]

[a] Geomicrobiology & Microbial Ecology, Center for Applied Geosciences, University of Tuebingen, Tuebingen, Germany
[b] Fertilization and Soil Matter Dynamics, Institute of Crop Science, University of Hohenheim, Stuttgart, Germany
[c] Plant Ecology and Ecotoxicology, Institute of Landscape and Plant Ecology, University of Hohenheim, Stuttgart, Germany
[d] Institute of Climate-Smart Agriculture, Johann Heinrich von Thünen-Institut, Federal Research Institute for Rural Areas, Forestry and Fisheries, Braunschweig, Germany
[e] Department of Soil Physics, Helmholtz Centre for Environmental Research – UFZ, Halle/Saale, Germany
[f] Department of Civil, Environmental, and Geo-Engineering, University of Minnesota, Minneapolis, MN, USA
[g] BioTechnology Institute, University of Minnesota, St. Paul, MN, USA

Supplementary Information

* Corresponding author. Tel.: +1-612-626-8225 and +1-612-624-8454.

E-mail address: sbehrens@umn.edu (S. Behrens).

1. Material and Methods

1.1 Soil characterization methods

Soil pH was determined in a 1:5 water suspension according to ISO 10390. Soil particle size distribution was determined according to ISO 11277 by sieving and sedimentation using different sieves and a Sedigraph III (Micromeritics, Norcross, GA, USA). $CaCO_3$ content was determined using a Calcimeter (Eijkelkamp, Giesbeek, The Netherlands) according to ISO 10693. Carbon and nitrogen were quantified according to ISO 10694 and 13878 using a Vario EL elemental analyser (Elementar, Hanau, Germany). For the quantification of the other elements listed in table 1 soil samples were digested prior to analysis. Samples were acid digested by microwave pressure digestion using a MLS Start 1500 microwave (MLS, Leutkirch, Germany) according to the manufacturer recommendations for soil. The resulting solution was analysed by Inductively Coupled Plasma Optical Emission Spectroscopy (ICP-OES) with an Optima 5300 DV (PerkinElmer, Waltham, MA, USA).

1.2 Gross nitrification and NO_3^- consumption rates

Gross nitrification and NO_3^- consumption rates were determined using the equations (1 - 2) provided by Davidson et al. 1991.

$$N = \frac{M_0 - M_1}{t} \times \frac{log(H_0M_1 / H_1M_0)}{log(M_0 / M_1)} \quad (1)$$

$$C = \frac{M_0 - M_1}{t} \times \frac{log(H_0 / H_1)}{log(M_0 / M_1)} \quad (2)$$

N : nitrification rate (mg N kg^{-1} dry soil d^{-1})

C : NO_3^- consumption rate (mg N kg^{-1} dry soil d^{-1})

M_0 : $^{14+15}NO_3^-$ pool at first time point (mg NO_3^--N kg^{-1} dry soil)

M_1 : $^{14+15}NO_3^-$ pool at second time point (mg NO_3^--N kg^{-1} dry soil)

H_0 : $^{15}NO_3^-$ pool at first time point (mg NO_3^--N kg^{-1} dry soil)

H_1 : $^{15}NO_3^-$ pool at second time point (mg NO_3^--N kg^{-1} dry soil)

t : time between first and second time point (days)

1.3 Quantitative polymerase chain reaction (qPCR) analyses

1.3.1 Nucleic acid extraction efficiencies

Nucleic acid extraction efficiencies were calculated based on the internal RNA and DNA standards using equation (3).

$$\textit{Extraction efficiency} = \frac{\textit{internal standard copies quantified in the final DNA or cDNA extract}}{\textit{DNA or RNA internal standard copies added prior to extraction}} \quad (3)$$

1.3.2 Quantitative polymerase chain reaction (qPCR)

Details about the used primers, reactions mixtures, thermal profiles and qPCR efficiencies are listed in table S1 and S2.

Table S1: Primers used for qPCRs.

target gene	primer name	primer sequence (5' - 3')	fragment size (bp)	reference
napA	V17m	TGGACVATGGGYTTYAAYC	152	Bru et al. (2007)
	napA4r	ACYTCRCGHGCVGTRCCRCA		
narG	narG-f	TCGCCSATYCCGGCSATGTC	173	Bru et al. (2007)
	narG-r	GAGTTGTACCAGTCRGCSGAYTCSG		
nirK	nirK876c	ATYGGCGGVCAYGGCGA	164	Henry et al. (2004) (modified)
	nirK1040	GCCTCGATCAGRTTRTGGTT		
nirS	nirSCd3aF	AACGYSAAGGARACSGG	407	Kandeler et al. (2006)
	nirSR3cd	GASTTCGGRTGSGTCTTSAYGAA		
typical *nosZ*	nosZ2F	CGCRACGGCAASAAGGTSMSSGT	267	Henry et al. (2006)
	nosZ2R	CAKRTGCAKSGCRTGGCAGAA		
atypical *nosZ*	nosZ-II-F	CTNGGNCCNYTKCAYAC	698	Jones et al. (2013)
	nosZ-II-R	GCNGARCARAANTCBGTRC		
Internal standard	APA9F	CGAACCTGGACTGTTATGATG	87	Thonar et al. (2012)
	APA9R	AATAAACAATCCCCTGTATTTCAC		

nirK876c was modified from nirK876 (Henry et al., 2004) to increase binding specificity

Table S2: Details about the qPCR assays performed with DNA and cDNA extracts.

target gene	origin of standard	reaction mixture	volume (µl)	thermal profile	Efficiency [%]
napA	*Pseudomonas aeruginosa* PAO1	SsoAdvanced Universal SYBR<br>V17m (5 µM)<br>napA4r (5 µM)<br>PCR water<br>Template	5<br>0.5<br>0.5<br>3<br>1	98°C – 15 s<br>55°C – 15 s<br>72°C – 15 s<br>X 45 cycles	74
narG	*Pseudomonas aeruginosa* PAO1	SsoAdvanced Universal SYBR<br>narG-f (5 µM)<br>narG-r (5 µM)<br>PCR water<br>Template	5<br>0.5<br>0.5<br>3<br>1	98°C – 10 s<br>62°C – 20 s<br>X 40 cycles	84
nirK	*Ensifer meliloti* 1021	SsoAdvanced Universal SYBR<br>nirK876c (5 µM)<br>nirK1040 (5 µM)<br>PCR water<br>Template	5<br>0.5<br>0.5<br>3<br>1	98°C – 10 s<br>58°C – 20 s<br>X 40 cycles	92
nirS	*Ralstonia eutropha* H16	SsoAdvanced Universal SYBR<br>nirSCd3aF (5 µM)<br>nirSRcd (5 µM)<br>PCR water<br>Template	5<br>1<br>1<br>2<br>1	98°C – 30 s<br>57°C – 30 s<br>72°C - 30 s<br>X 40 cycles	89
typical *nosZ*	*Ensifer meliloti* 1021	SsoAdvanced Universal SYBR<br>nosZ2F (5 µM)<br>nosZ2R (5 µM)<br>PCR water<br>Template	5<br>0.5<br>0.5<br>3<br>1	98°C – 15 s<br>60°C – 25 s<br>X 40 cycles	74
atypical *nosZ*	*Gemmatimonas aurantiaca* T-27	IQ SYBR<br>nosZ-II-F (5 µM)<br>nosZ-II-R (5 µM)<br>PCR water<br>Template	5<br>2<br>2<br>0<br>1	98°C – 30 s<br>54°C – 30 s<br>72°C - 45 s<br>80°C – 30 s<br>X 40 cycles	86
Internal standard	*African cassava mosaic virus*-[Nigeria-Ogo]	SsoAdvanced Universal SYBR<br>APA9F (5 µM)<br>APA9R (5 µM)<br>PCR water<br>Template	5<br>0.5<br>0.5<br>3<br>1	98°C – 10 s<br>50°C – 15 s<br>X 35 cycles	89

1.4 N_2O source partitioning and rates of NO_3^--derived N_2O and N_2

1.4.1 Fractions of NO_3^--derived N_2O and N_2

For each gas sample collected in experiment 1, 2, and 3, the fraction of N_2 and/or N_2O evolving from the ^{15}N-labeled NO_3^- pool (f_p), was calculated using the equations provided by (Spott et al., 2006).

$$f_p = \frac{a_m - a_{bgd}}{a_p - a_{bgd}} \tag{4}$$

a_{bgd} : ^{15}N abundance of atmospheric background

a_m : measured ^{15}N abundance of N_2 and N_2O

$$a_m = \frac{^{29}R + 2 \times {}^{30}R}{2\left(1 + {}^{29}R + {}^{30}R\right)} \tag{5}$$

a_p : calculated ^{15}N abundance of active ^{15}N-labeled NO_3^- pool

$$a_p = \frac{^{30}x_m - a_{bgd} \times a_m}{a_m - a_{bgd}} \tag{6}$$

$^{30}x_m$: measured fraction of *m/z* 30 in N_2 and converted N_2O

$$^{30}x_m = \frac{^{30}R}{1 + {}^{29}R + {}^{30}R} \tag{7}$$

In experiment 1, nitrogen isotope ratios of N_2O were determined by analysis of intact N_2O molecules ($^{45}R = (^{14}N^{15}N^{16}O + {}^{15}N^{14}N^{16}O + {}^{14}N^{14}N^{17}O)/{}^{14}N^{14}N^{16}O$; $^{46}R = (^{15}N^{15}N^{16}O + {}^{14}N^{14}N^{18}O)/{}^{14}N^{14}N^{16}O$). To use the previous equations, isotope ratios (^{45}R and ^{46}R) were oxygen-corrected according to (Bergsma et al., 2001) using equations (8) and (9).

$$^{29}R = {}^{45}R - {}^{17}R \tag{8}$$

$$^{30}R = {}^{46}R - {}^{29}R \times {}^{17}R - {}^{18}R \tag{9}$$

For ^{17}R and ^{18}R we used the values suggested by (Bergsma et al., 2001):

^{17}R = 0.000373, ^{18}R = 0.0020052

1.4.2 Rates of NO_3^--derived N_2O and N_2

Concentrations of NO_3^--derived N_2O and N_2 (c_p) in ppm were calculated according to equation (10):

$$c_p = f_p \times c_H \quad (10)$$

c_H: headspace concentration (ppm) of total N_2O (GC-ECD, experiment 1) or total N_2 (N_2 concentration of artificial gas mixture = 20 000 ppm, assuming a negligible relative increase in N_2 concentration due to microbial N_2 production, experiment 2 and 3).

In experiment 2 and 3, N_2O and N_2 in the headspace was diluted during each sampling occasion due to gas exchange between the headspace and the sample vial initially filled with the artificial gas mixture. Diluted NO_3^--derived N_2O and N_2 concentrations were corrected using equations (11), (12), and (13). No dilution correction was needed for experiment 1 due to the different sampling strategy.

$$cc_{p1} = c_{p1} \times \left(1 + \frac{V_{S1}}{V_{T1}}\right) \quad (11)$$

$$cc_{p3} = c_{p3} \times \left(1 + \frac{V_{S1}}{V_{T1}}\right) \times \left(1 + \frac{V_{S3}}{V_{T3}}\right) \quad (12)$$

$$cc_{ps} = c_{ps} \times \left(1 + \frac{V_{S1}}{V_{T1}}\right) \times \left(1 + \frac{V_{S3}}{V_{T3}}\right) \times \left(1 + \frac{V_{Ss}}{V_{Ts}}\right) \quad (13)$$

$cc_{p1,3,s}$: corrected NO_3^--derived N_2O and N_2 concentrations (ppm) in samples collected after 1 h of enrichment (cc_{p1}), 3 h of enrichment (cc_{p3}), and after shaking (cc_{ps})

$c_{p1,3,s}$: NO_3^--derived N_2O and N_2 concentrations (ppm) in samples collected after 1 h of enrichment (c_{p1}), 3 h of enrichment (c_{p3}), and after shaking (c_{ps})

$V_{S1,3,s}$: volume of the gas samples during sampling after 1 h of enrichment (V_{S1}), 3 h of enrichment (V_{S3}), and after shaking (V_{Ss})

$V_{T1,3,s}$: total volume (headspace and gas sample) during sampling after 1 h of enrichment (V_{T1}), 3 h of enrichment (V_{T3}), and after shaking (V_{Ts})

In experiment 1, NO_3^--derived N_2O emission rates (ER_p) were calculated according to equation (14). As the concentration of NO_3^--derived N_2O after 0 h of enrichment was 0, only values determined from samples collected after 1 h (c_{p1}) were considered for emission rate calculation. For the determination of NO_3^--derived N_2O and N_2 emission rates in experiment 2 and 3 equation (15) was used.

$$ER_p = c_{p1} \times \frac{k \times V_H}{m} \quad (14)$$

$$ER_p = \frac{\left(cc_{p3} - cc_{p1}\right)}{t} \times \frac{k \times V_H}{m} \quad (15)$$

ER_p : emission rates of NO_3^--derived N_2O and N_2 (mg N_2O-N kg^{-1} dry soil h^{-1})

t : time (h) between first (after 1 h) and second sampling (after 3 h)

k : unit conversion factor calculated as $k = \frac{molar\ mass\ of\ N\ in\ N_2O\ and\ N_2}{molar\ volume\ of\ gas\ (20°C)} = \frac{28.014}{24.055}$

V_H : volume of the headspace during sampling (equal after 1 h and 3 h of enrichment)

m : mass of dry soil (g) in the gas enrichment container

The NO_3^--derived N_2O and N_2 soil entrapment/emission ratio (SE_p / E_p), defined as the concentration (ppm) of NO_3^--derived N_2O and N_2 accumulating in the headspace before and after shaking at day 2 in experiment 3 was calculated using equation (16)

$$SE_p / E_p = \frac{cc_{ps.d2} - cc_{p3.d2}}{cc_{p3.d2}} \quad (16)$$

$cc_{p3.d2,s.d2}$: corrected NO_3^--derived N_2O and N_2 concentrations (ppm) in samples collected after 3 h of enrichment ($cc_{p3.d2}$), and after shaking ($cc_{ps.d2}$) at day 2

NO_3^--derived N_2O and N_2 soil entrapment (SER_p) and total production (TPR_p) rates were calculated from samples collected during experiment 3 after 2 days of incubation using equations (17) and (18), respectively.

$$SER_p = ER_{p.d2} \times SE_p / E_p \quad (17)$$

$$TPR_p = ER_{p.d2} + SER_p \quad (18)$$

SER_p : soil entrapment rates of NO_3^--derived N_2O and N_2 (mg N_2O-N kg^{-1} dry soil h^{-1}) at day 2

$ER_{p.d2}$: emission rates of NO_3^--derived N_2O and N_2 (mg N_2O-N kg^{-1} dry soil h^{-1}) at day 2

TPR_p : total production rates of NO_3^--derived N_2O and N_2 (mg N_2O-N kg^{-1} dry soil h^{-1}) at day 2

1.4.3 N_2O source partitioning

The contribution of NO_3^--derived N_2O to total soil-derived N_2O emissions ($f_{nitrate}$) was calculated from samples collected during experiment 1 using equation (19).

$$f_{nitrate} = \frac{f_p}{f_{soil}} \quad (19)$$

$f_{nitrate}$: fraction of NO_3^--derived N_2O of total soil-derived N_2O

f_p : fraction of N_2O evolving from the ^{15}N-labeled NO_3^- pool

f_{soil} : fraction of N_2O evolving from soil calculated as: $f_{soil} = \frac{total\ N_2O - background\ N_2O}{total\ N_2O}$

2. Results

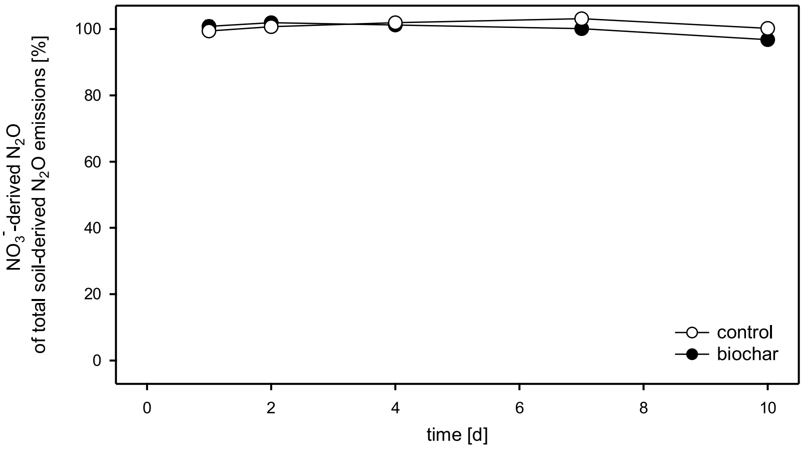

Figure S1: Contribution of NO_3^--derived N_2O to total soil-derived N_2O emissions in control (open circles) and biochar (solid circles) over time.

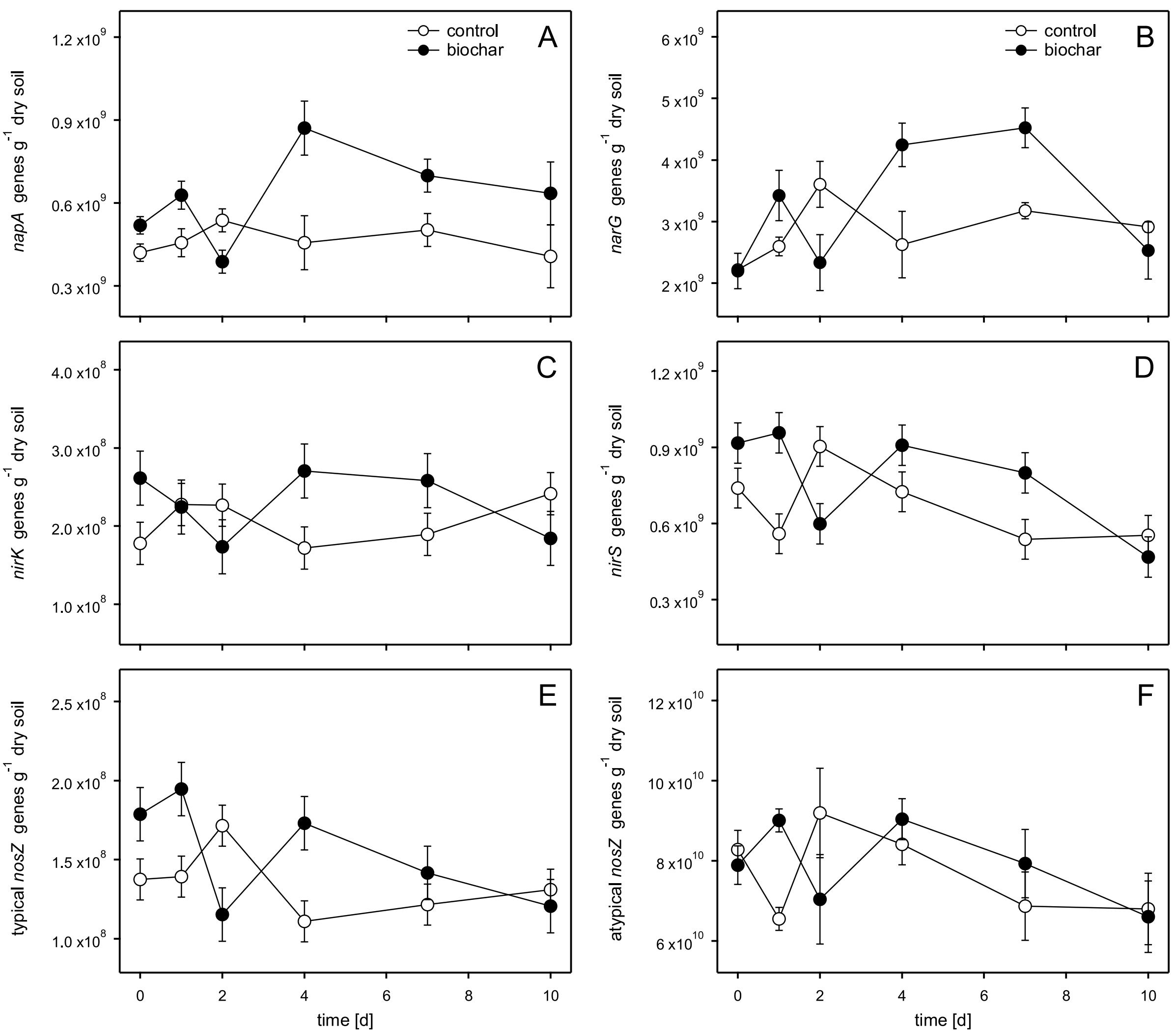

Figure S2: Gene copy numbers of functional marker genes of denitrification in control (open circles) and biochar (solid circles) microcosms over time. The different panels show: (A) *napA* (B) *narG*, (C) *nirK*, (D) *nirS*, (E) typical *nosZ*, and (F) atypical *nosZ*. Data points and error bars represent means and standard errors (n=3), respectively.

Table S3: Results from two-way ANOVAs for the gene data. The table shows F-statistics and p-values for the main effects "biochar" and "time" and their interaction "biochar*time".

**parameter**	**biochar**		**time**		**biochar * time**	
	F	p	F	p	F	p
napA genes	7.18	**0.026**	2.21	0.154	2.98	0.084
narG genes	0.82	0.387	5.49	0.065	2.06	0.256
nirK genes	0.95	0.340	0.16	0.975	2.23	0.086
nirS genes	3.02	0.096	3.36	**0.020**	3.73	**0.013**
typical *nosZ* genes	2.96	0.099	1.73	0.169	3.93	**0.010**
atypical *nosZ* genes	0.07	0.799	1.76	0.232	3.02	0.084

References

Bergsma, T.T., Ostrom, N.E., Emmons, M., and Robertson, G.P. (2001) Measuring simultaneous fluxes from soil of N_2O and N_2 in the field using the ^{15}N-gas "nonequilibrium" technique. *Environmental Science & Technology* **35**: 4307-4312.

Bru, D., Sarr, A., and Philippot, L. (2007) Relative abundances of proteobacterial membrane-bound and periplasmic nitrate reductases in selected environments. *Applied and Environmental Microbiology* **73**: 5971-5974.

Henry, S., Bru, D., Stres, B., Hallet, S., and Philippot, L. (2006) Quantitative detection of the *nosZ* gene, encoding nitrous oxide reductase, and comparison of the abundances of 16S rRNA, *narG*, *nirK*, and *nosZ* genes in soils. *Applied and Environmental Microbiology* **72**: 5181-5189.

Henry, S., Baudoin, E., Lopez-Gutierrez, J.C., Martin-Laurent, F., Baumann, A., and Philippot, L. (2004) Quantification of denitrifying bacteria in soils by nirK gene targeted real-time PCR. *Journal of Microbiological Methods* **59**: 327-335.

Jones, C.M., Graf, D.R., Bru, D., Philippot, L., and Hallin, S. (2013) The unaccounted yet abundant nitrous oxide-reducing microbial community: a potential nitrous oxide sink. *Isme Journal* **7**: 417-426.

Kandeler, E., Deiglmayr, K., Tscherko, D., Bru, D., and Philippot, L. (2006) Abundance of *narG*, *nirS*, *nirK*, and *nosZ* genes of denitrifying bacteria during primary successions of a glacier foreland. *Applied and Environmental Microbiology* **72**: 5957-5962.

Spott, O., Russow, R., Apelt, B., and Stange, C.F. (2006) A ^{15}N-aided artificial atmosphere gas flow technique for online determination of soil N_2 release using the zeolite Kostrolith SX6. *Rapid Communications in Mass Spectrometry* **20**: 3267-3274.

Thonar, C., Erb, A., and Jansa, J. (2012) Real-time PCR to quantify composition of arbuscular mycorrhizal fungal communities--marker design, verification, calibration and field validation. *Molecular Ecology Resources* **12**: 219-232.
